# Methyl group assignment using pseudocontact shifts with PARAssign

**DOI:** 10.1007/s10858-017-0136-3

**Published:** 2017-11-27

**Authors:** Mathilde Lescanne, Simon P. Skinner, Anneloes Blok, Monika Timmer, Linda Cerofolini, Marco Fragai, Claudio Luchinat, Marcellus Ubbink

**Affiliations:** 1 Leiden Institute of Chemistry, Leiden University, Einsteinweg 55, 2333 CC Leiden, The Netherlands; 2 Department of Molecular and Cell Biology, Leicester Institute for Structural- and Chemical Biology, University of Leicester, Lancaster Road, Leicester, LE1 7RH UK; 3 Present Address: School of Molecular and Cellular Biology, Faculty of Biological Sciences & Astbury Centre for Structural Molecular Biology, University of Leeds, Leeds, LS2 9JT UK; 4 Giotto Biotech, Via Madonna del Piano, 6, 50019 Sesto Fiorentino, FI Italy; 5 Magnetic Resonance Center - CERM, University of Florence, Via Sacconi 6, 50019 Sesto Fiorentino, FI Italy

**Keywords:** Assignment, Pseudocontact shift, Methyl groups, NMR spectroscopy, Paramagnetic tag, Heat shock protein

## Abstract

A new version of the program PARAssign has been evaluated for assignment of NMR resonances of the 76 methyl groups in leucines, isoleucines and valines in a 25 kDa protein, using only the structure of the protein and pseudocontact shifts (PCS) generated with a lanthanoid tag at up to three attachment sites. The number of reliable assignments depends strongly on two factors. The principle axes of the magnetic susceptibility tensors of the paramagnetic centers should not be parallel so as to avoid correlated PCS. Second, the fraction of resonances in the spectrum of a paramagnetic sample that can be paired with the diamagnetic counterparts is critical for the assignment. With the data from two tag positions a reliable assignment could be obtained for 60% of the methyl groups and for many of the remaining resonances the number of possible assignments is limited to two or three. With a single tag, reliable assignments can be obtained for methyl groups with large PCS near the tag. It is concluded that assignment of methyl group resonances by paramagnetic tagging can be particularly useful in combination with some additional data, such as from mutagenesis or NOE-based experiments. Approaches to yield the best assignment results with PCS generating tags are discussed.

**Electronic supplementary material:**

The online version of this article (doi:10.1007/s10858-017-0136-3) contains supplementary material, which is available to authorized users.

## Introduction

Liquid state NMR is widely used to study protein structure, dynamics and interactions with atomic detail. Resonance assignment is a prerequisite for all these studies. Methyl groups have become important in biomolecular NMR to probe biomolecular interactions (Wiesner and Sprangers 2015) as well as dynamics of proteins over a large range of timescales, from picoseconds to seconds (Sprangers and Kay 2007). Furthermore, the hydrophobic nature of methyl groups makes them suitable to probe the core of a protein (Tugarinov and Kay 2005) and study specific interactions it may have with other proteins, ligands or substrates (Gross et al. 2003). From a spectroscopic point of view, ^13^CH_3_ methyl groups are valuable probes in very large systems because their inherent fast rotation and geometry give them favorable relaxation properties that make methyl groups suitable for TROSY experiments (Pervushin et al. 1997; Tugarinov et al. 2003). Specific isotope labeling on isoleucine, leucine and valine (ILV) methyl groups is an established approach (Tugarinov et al. 2006) to reduce spectral crowding and to provide good spatial coverage due to dispersion of these residues through the protein. Assignment of ILV methyl groups is, however, non-trivial. To take advantage of standard *J*-coupling based assignments procedures, uniformly labeled samples are needed, requiring the production of additional samples and time-consuming 3D NMR experiments. For large systems, methyl assignment is further complicated by the need for fractional or complete deuteration of the other hydrogen positions to reduce relaxation by dipolar couplings. Various approaches have been published to assign methyl groups in large proteins. Systematic site-directed mutagenesis, combined with stereospecific labeling on methyl groups has been applied to TET2 (Amero et al. 2011; Mas et al. 2013). To circumvent the size limitation, the so-called “divide and conquer” approach also proved to be effective. Here, individual domains or subunits are first assigned, followed by a transfer of assignments to the spectrum of the larger multidomain protein or multisubunit protein complex (Mas et al. 2013; Gelis et al. 2007; Velyvis et al. 2009). Another approach is to use multidimensional NOESY spectra in combination with a crystal structure (Venditti et al. 2011). This approach has been implemented in the MAP-XSII software (Xu and Matthews 2013) that automatically assigns methyl groups based on a known structure combined with NOEs measured on 4D NOESY experiments. Similarly, the program FLAMEnGO uses a combination of 3D NOESY, methine–methyl TOCSY and paramagnetic relaxation enhancements (PREs) and a protein structure to assign methyl groups (Chao et al. 2014).

More generally, paramagnetic NMR has become attractive for assignment (Pan et al. 2016), and software packages have been developed such as Platypus (Pintacuda et al. 2004), Echidna (Schmitz et al. 2006), Possum (John et al. 2007) and PARAssign (Skinner et al. 2013). Platypus and Echidna have been used to assign NH resonances on the basis of a crystal-structure and pseudo-contact shifts (PCS). Possum assigns methyl groups with PCS measured with paramagnetic centers for which the magnetic susceptibility tensor is known. This requires assignment of at least five amides or methyl groups to be available. PARAssign does not require any assignment or prior knowledge of the susceptibility tensors. It automatically assigns ^1^H, ^13^C, or ^15^N resonances based on PCS from several paramagnetic centers obtained from 2D HSQC or TROSY spectra and a 3D structure of the protein and it also provides an assessment of the reliability of the assignment. It has been shown that PARAssign software could assign the amides of pseudoazurin (PAZ) (125 residues) using PCS from three different paramagnetic centers. PARAssign has been tested also on P450cam (414 residues) for methyl groups assignment using synthetic data (Skinner et al. 2013).

Here, we evaluate the ability of PARAssign to assign the 76 ILV methyl groups of a 25 kDa protein, the N-terminal domain of HSP90 (ntd-HSP90), using experimentally observed PCS. Ntd-HSP90 (Pearl 2016; Karagoz et al. 2011; Li et al. 2012a) is a well-studied chaperone protein, for which methyl group assignments are available (Shah et al. 2012). PCS were induced by the paramagnetic tag CLaNP-5, (Keizers et al. 2008) attached to the protein via disulfide linkages to engineered cysteine residues. We find that, using two judiciously placed paramagnetic tags, PARAssign can assign up to 60% of the 76 methyl groups of ntd-HSP90 with high reliability and give many correct suggestions for the remaining 40%. The quality of the input data is an important determinant for the number of reliable assignments. With a single tag, reliable assignments can be obtained for nearby nuclei with large PCS.

## Materials and methods

### Protein preparation

Four double cysteine mutants of the ntd-HSP90 were designed on the surface of the protein, S50C/D54C, A101C/N105C, T149C/I187C, and M130C/Q133C. Ntd-HSP90 does not have any native cysteines. The protein was produced by overexpression of the gene for ntd-HSP90 (residues 8-233, UNIPROT entry P07900) with a cleavable His_6_ tag in the PQTEV (Scheich et al. 2004) vector in *Escherichia coli* BL21 DE3. The plasmid was kindly provided by Dr Dipen Shah. Cultures of 500 mL M9 medium in 2 L Erlenmeyer flaks were incubated at 37 °C at 150 rpm. At an OD_600_ of 0.6, IPTG (1 mM) was added to induce gene expression, the temperature was reduced to 18 °C and the incubation was continued overnight. For ^15^N labeled protein, ^15^N ammonium chloride (0.3 g/L) was added as sole nitrogen source. For ^13^CH_3_ methyl labeling, two samples were produced labeled either at Leu-δ1-δ2/Val-γ1-γ2-[^13^CH_3_] or Ile-δ1-[^13^CH_3_]. Consequently, it was possible to identify unambiguously the residue type of the Ile methyl groups. Either 50 mg/L 2-keto-3,3-1,2,3,4-^13^C-butyrate (Ile ^13^CH_3_ labeling) or 100 mg/L of 2-keto-3-methyl-3-1,2,3,4-^13^C butyrate (Leu and Val ^13^CH_3_ labeling) were added to the M9 medium with 4 g/L ^12^C glucose, one hour before induction at OD_600_ ~ 0.5. The cells were harvested by centrifugation at 6000 rpm for 20 min at 4 °C. Cell lysis was performed using lysozyme. PMSF and DNAse were added for protease inhibition and DNA breakdown. Protein purification was performed with a His-trap NTA-column, eluting with an imidazole gradient (5–500 mM imidazole in 100 mL). The His-tag was then cleaved with TEV protease during overnight incubation at 4 °C. The His-tag and TEV protease were removed by a second elution from the column.

CLaNP-5 was synthesized as described before (Keizers et al. 2008). For CLaNP-5 attachment, the protein was reduced for 1 h on ice in the presence of 5 mM DTT. Then, DTT was removed with a Sephadex G-25 PD10 desalting column (GE Healthcare) and the protein solution was mixed immediately with four molar equivalents of either Yb^3+^-CLaNP-5 or Lu^3+^-CLaNP5 and incubated at room temperature for 1 h. The sample was loaded on an analytical Superdex 200 gel filtration column (GE Healthcare) to remove excess CLaNP-5 and protein dimers.

### NMR spectroscopy

NMR samples contained 50–100 μM protein in 50 mM Tris–HCl, 100 mM NaCl, pH 7.5 and, 6% D_2_O. All the spectra were recorded at a temperature of 298 K. ^15^N-HSQC spectra were recorded on a Bruker Ascend Avance III 850 HD MHz spectrometer, equipped with a TCI cryoprobe, with spectral widths of 20.00 ppm (17.00 kHz) and 35.00 ppm (3.015 kHz) in the ^1^H and ^15^N dimensions, respectively. Leu-δ1-δ2/Val-γ1-γ2-[^13^CH_3_] and Ile-δ1-[^13^CH_3_] protein spectra were recorded on Bruker 800 and 850 MHz spectrometers equipped with TXI cryoprobes, with a spectral width of 20.00 ppm in the ^13^C dimension. Experiments were performed with 16 or 32 scans. Data were processed with Topspin 3.2 and NMRpipe 8.2 (Delaglio et al. 1995) and spectra were analyzed with CCPNMR Analysis 2.4 (Vranken et al. 2005).

### Determination of the PRE-derived cutoff distance

The cutoff distance below which a signal would be too broad to be detected because of PRE was calculated using Eq. 1. The line broadening due to PRE (Г_2_) was estimated to render nuclei within 9 Å from the Yb^3+^ ion invisible, based on the expected Curie relaxation (Eq. 1) (Bertini et al. 2015), which is the dominant transverse relaxation mechanism for ^1^H by lanthanoids with an anisotropic magnetic susceptibility.

equation 1 $${r_{cutoff}}=\sqrt[6]{{\frac{1}{{5{\varGamma _2}}}{{\left( {\frac{{{\mu _0}}}{{4\pi }}} \right)}^2}\frac{{\omega _{H}^{2}{{\left( {{g_e}{\mu _B}} \right)}^4}{J^2}{{(J+1)}^2}}}{{{{\left( {3{k_B}T} \right)}^2}}}\left( {4{\tau _r}+\frac{{3{\tau _r}}}{{1+\omega _{H}^{2}\tau _{r}^{2}}}} \right)}}$$

Here, *μ*_0_ is the permeability of a vacuum, *ω*_*H*_ is the Larmor frequency of the hydrogen nucleus, g_e_ is the Landé *g-*factor, *μ*_*B*_ is the Bohr magneton, *J* is the total spin quantum number of the paramagnetic lanthanoid, *k*_*B*_ is the Boltzmann constant, *T* is the absolute temperature, and *τ*_*r*_ is the rotational correlation time of the molecule.

### Methyl group assignments

Methyl group assignments from PARAssign were compared to those obtained with traditional 3D NMR procedures. The published methyl group assignments (Shah et al. 2012) were checked and completed using CccoNH/HccoNH/HSQC spectra, kindly provided by Dr. Dipen Shah and Dr. Eiso Ab.

### Assignment procedure

The PARAssign code has been refactored for the sake of readability and easier maintenance. A new reliability criterion has been implemented (see “Results and discussion” section). The core procedure remains unchanged (Skinner et al. 2013). The PARAssign user must supply the PCS file containing all the measured proton PCS, a PDB file and the JSON configuration file with information about the assignment to be performed (atom names, stereospecificity of the input, double Cys mutation sites, starting point for the tensor parameters, input file directory, output file directory). ^13^C PCS were not considered as input, because the resolution in this dimension is lower and the residual chemical shift anisotropy is significant for carbon but can be neglected for protons. The PARAssign output includes the assignment plot, tensor parameters, tensor PDB file(s) and predicted PCS for all the assignable groups in the structure. The PARAssign input consisted of the ntd-HSP90 structure, PDB entry 3T0Z (Li et al. 2012b), and the PCS datasets acquired for the S50C/D54C, A101C/N105C and T149C/I187C mutants tagged with Lu^3+^-CLaNP-5 as diamagnetic control and Yb^3+^-CLaNP-5 as the paramagnetic center. Although we used two different samples for isoleucines and leucines + valines, PARAssign can select the predicted type(s) of each assignable peak by entering the residue type code in the input file (‘ILE’ or ‘VAL LEU’ in our case but all possible combinations are acceptable). A run of PARAssign comprises an iteration of two consecutive procedures, the assignment procedure and the tensor optimization procedure using PCS calculated during the previous assignment procedure. In the assignment procedure of the PARAssign software, the Hungarian method for minimal cost assignment (Kuhn 1955) is used to perform the assignment of the input PCS, using a scoring factor cost function to populate the assignment matrix:

equation 2 $$Scoring~factor=\frac{1}{{Pc}} \times \mathop \sum \limits_{{i=1}}^{{Pc}} \sqrt {\frac{{{{\left( {\delta _{{PCS,i}}^{{pred}} - \delta _{{PCS,i}}^{{exp}}} \right)}^2}}}{{{{\left( {\left|\delta _{{PCS,i}}^{{pred}}\right|+\left|\delta _{{PCS,i}}^{{exp}}\right|} \right)}^2}}}}$$

where Pc is the number of paramagnetic centers. PARAssign attributes a scoring function of 1 to assignments for which experimental and predicted PCS have opposite signs, thus disfavoring the assignment. A scoring factor threshold is user-defined (default 0.2) and is used to filter out assignments for the optimization procedure for which the scoring factor is too high. A high threshold yields many assignments with poor accuracy and a low threshold yields few assignments. The optimal threshold is the lowest possible value that yields the maximum number of assignments. For instance, when starting with a scoring factor of 0.7 and decreasing it progressively, the optimum threshold is passed when the number of assigned groups starts to drop. The tensor parameters optimization procedure consists of the minimization of the square difference between calculated and experimental PCS for all paramagnetic centers. This sequential least squares programming procedure (Kraft 1988) is performed by the python function scipy.optimize.fmin_slsqp (http://www.scipy.org). The output of PARAssign gives the susceptibility tensors of the paramagnetic centers and the assignment of the diamagnetic protein spectrum with a reliability scoring for each assignment (discussed below). The output figures included in this article may not reflect the true output quality. In the original .png image, the user can zoom in to read all the details. For this paper, it is relevant to be able to judge the quality of the assignment at a glance.

## Results and discussion

### Selection of tagging sites

Assignment based on PCS requires accurate PCS that are not affected by averaging effects due to tag mobility. For this reason, the two-armed caged lanthanoid NMR probe (CLaNP) #5 was used (Keizers et al. 2007, 2008), which requires two Cys residues located close together on the surface of the protein for attachment. Ntd-HSP90 has no Cys residues, so pairs of cysteines were engineered. The criteria for selection were (1) good distribution of the sites over the protein surface, (2) limited mobility of the attachment site by using secondary structure elements, (3) sufficiently exposed sites without steric hindrance of large side-chains. Four double cysteine mutations were designed, three of which are on α-helices (S50C/D54C, Cα–Cα = 6.0 Å; A101C/N105C, Cα–Cα = 5.9 Å; M130C/Q133C, Cα–Cα = 5.7 Å) and one was across neighboring strands of a beta-sheet (T149C/I187C, with Cα–Cα = 5.0 Å). Out of the four double cysteine variants, the three with the highest yields were selected, which were S50C/D54C, A101C/N105C and T149C/I187C, with yields of 30, 30 and 20 mg/L in minimal medium, respectively. Variant M130C/Q133C yielded only 1 mg/L and was not considered further.

### Reference Δχ-tensor determination using backbone amides

**Fig. 1:**
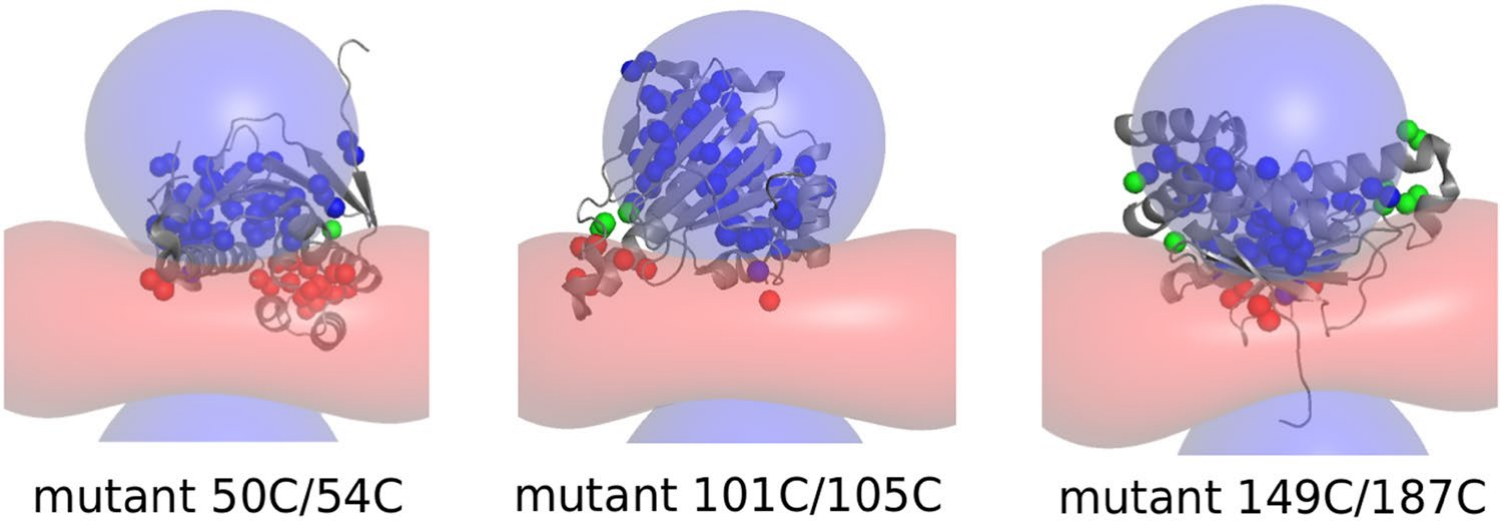
Predicted methyl group PCS. Methyl groups that are predicted to experience positive PCS, negative PCS and PCS smaller than 0.04 ppm are represented as blue, red and green spheres, respectively. The − 0.04 and 0.04 ppm isosurfaces of the Δχ-tensors based on the amide PCS are shown as red and blue surfaces

For reference, the lanthanoid location and the orientation of the Δχ-tensor were first determined on the basis of amide proton PCS and using Numbat software (Schmitz et al. 2008) with a crystal-structure of ntd-HSP90 [PDB entry 3T0Z (Li et al. 2012b)]. Diamagnetic assignments for the amide groups were available (Jacobs et al. 2006), BMRB entry 7003. The three mutants were ^15^N-labeled and tagged with the diamagnetic control tag Lu^3+^-CLaNP-5 or the paramagnetic tag Yb^3+^-CLaNP-5 and PCS were obtained from ^15^N-^1^H HSQC spectra. For mutants S50C/D54C and A101C/N105C, the agreement between observed and calculated PCS was excellent (Fig. S1). For mutant T149C/I187C the fit was of somewhat lower quality, for reasons discussed later. To check whether significant PCS could be expected for most methyl groups, PCS were predicted for the three Δχ-tensors derived from the amide PCS. Figure 1 shows that with Yb^3+^-CLaNP-5, PCS ≥ 0.04 ppm are expected for most methyl groups for each of the three tag locations. These amide based Δχ-tensors were used solely to generate the simulated data and for comparison with the ones refined by PARAssign for the experimental methyl group data, see the sections below.

### Tagging effects on methyl group spectra

**Fig. 2:**
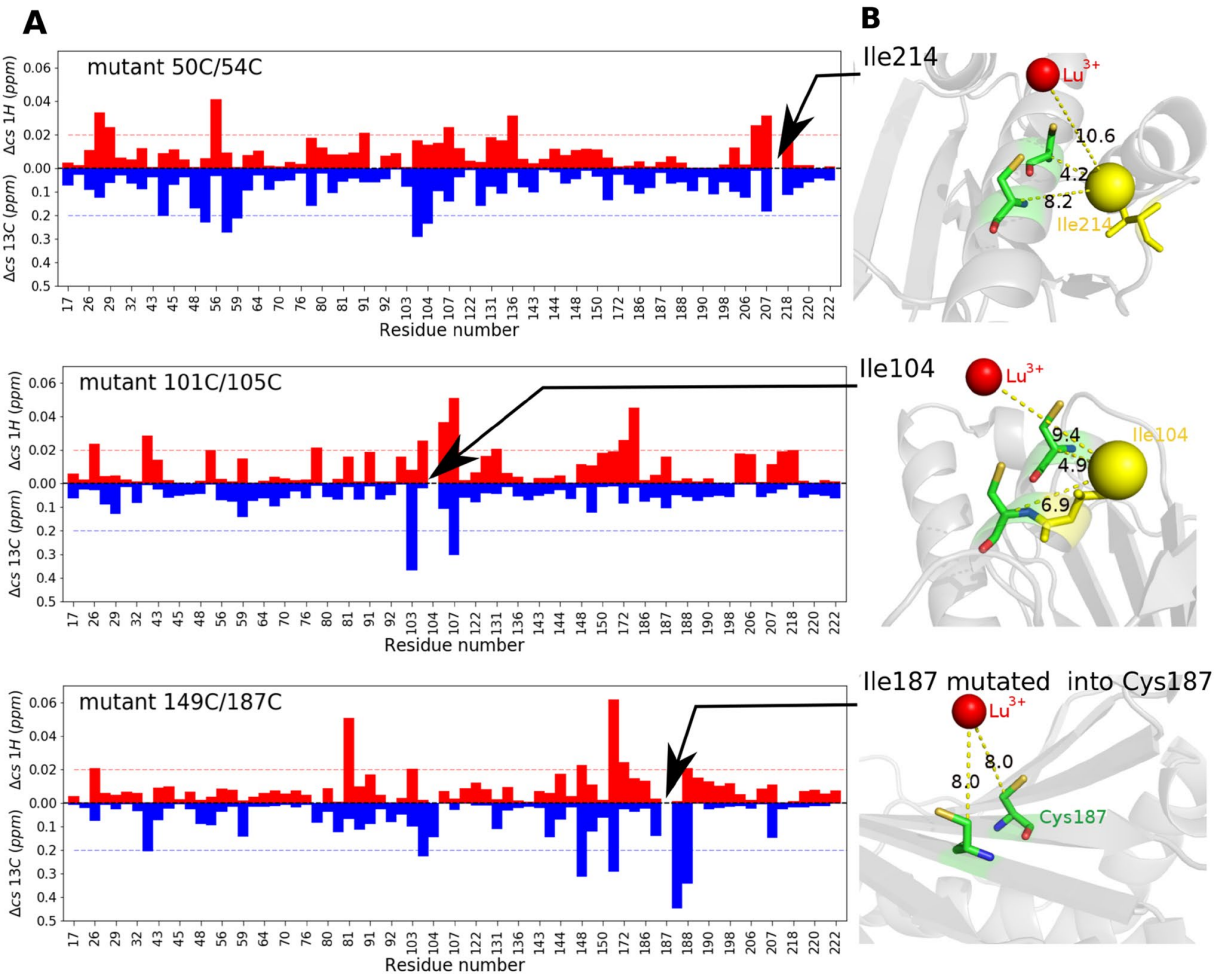
Mutation and tagging effects. **a** Absolute chemical shift differences between WT and Lu^3+^-tagged mutant ntd-HSP90 resonances for ^1^H (red) and ^13^C (blue) are plotted for each methyl group. The dotted lines mark significant changes, with 0.2 ppm for ^13^C and 0.02 for ^1^H. **b** Location of methyl groups with unmatched resonances relative to the tag site. Distances are expressed in Ångström

To establish the effects of a double cysteine mutation and the attachment of CLaNP-5 on methyl resonances, ^13^C-^1^H-HSQC spectra were acquired for the WT and Lu^3+^-CLaNP-5 tagged mutant ntd-HSP90 with ILV methyl-labeling. The peaks from the mutant protein were matched to those of the wild-type. Most peaks could be readily matched and showed only very small chemical shift changes (Fig. 2a). Two peaks, one for mutant 50C/54C and one for mutant 101C/105C, could not be matched with certainty to equivalent peaks in the spectrum of wild-type ntd-HSP90. These methyl groups were located close to the tag (Fig. 2b). In mutant 149C/187C one Ile was replaced by a Cys for attachment of the tag. The limited chemical shift perturbations between WT and mutant ntd-HSP90 indicate that no major structural changes occur because of the mutations and the tagging.

### The pairing problem

**Fig. 3:**
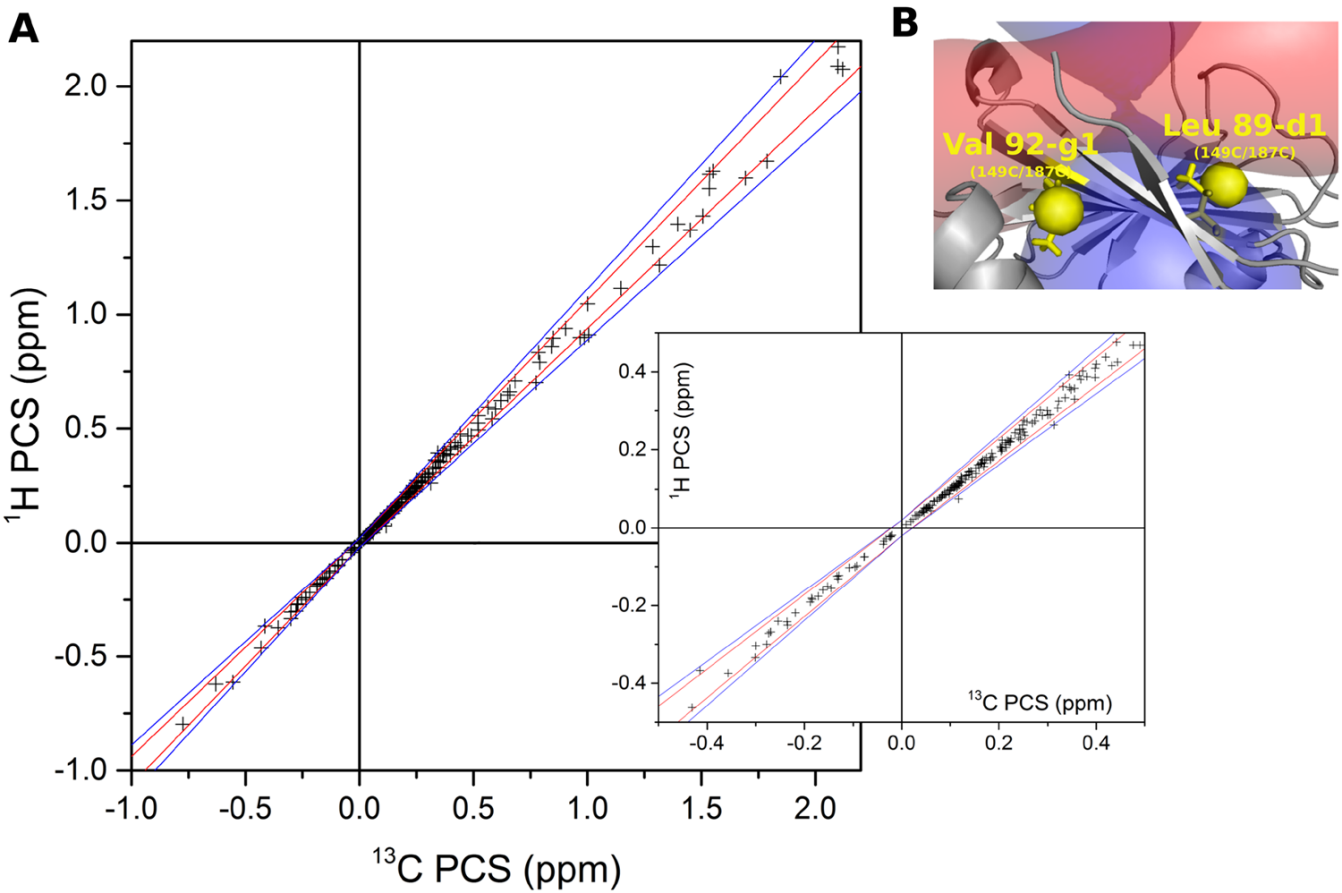
Correlation between the ^1^H PCS and ^13^C PCS of methyl groups. **a** The ^1^H and ^13^C methyl PCS predicted for the three tag positions on ntd-HSP90 are plotted. The PCS differing < 5% and 10% are contained between the red and blue lines, respectively. The inset shows a detail of the plot for small PCS. The lines intercept the axes at |0.02| ppm, taken as error margin. **b** Location of the two methyl groups, for the mutant 149C/187C, for which the deviation between ^1^H and ^13^C PCS is bigger than 10%. Isosurfaces are shown for PCS = 0.25 and − 0.25 ppm. Both methyl groups are very close to the region where the PCS changes sign

**Fig. 4:**
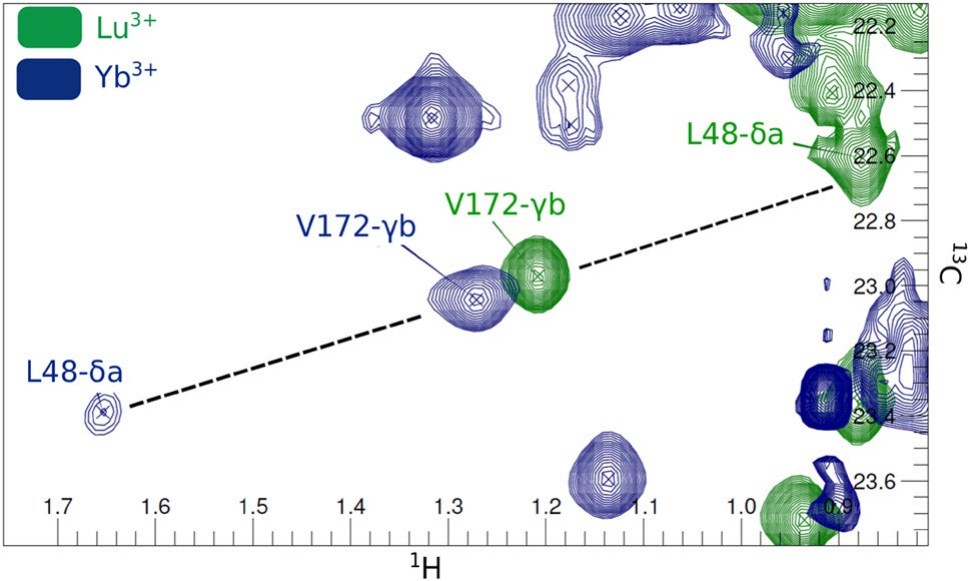
Illustration of the peak pairing problem. The peaks for V172 and L48 are on the same correlation line in the spectrum of Yb^3+^ tagged ntd-HSP90, making the pairing to the peaks in the spectrum of Lu^3+^ tagged protein problematic. In this case, the observation that the V172 in the paramagnetic state is more intense than the L48 peak in the diamagnetic state, suggests that those two cannot be paired

PCS are measured as differences in chemical shifts of resonances observed in a paramagnetic and the diamagnetic spectrum. The problem to be solved is thus to pair the diamagnetic and paramagnetic resonances belonging to the same methyl group. PCS expressed in ppm units do not depend on the gyromagnetic ratio of the observed nucleus. Consequently, when the distance between the nucleus and the paramagnetic center, as well as the orientation of the vector connecting them in the Δχ-tensor frame are similar for ^1^H and ^13^C, which is almost always true for a ^13^C^1^H_3_ methyl group, the ^13^C and ^1^H nuclei of a methyl group experience nearly the same PCS. In Fig. 3, the predicted PCS for all methyl ^13^C and ^1^H nuclei are plotted for ntd-HSP90 tagged at the three positions (224 points in total). The ^1^H PCS are averaged over the three PCS for the individual protons in the methyl group to account for the fast methyl rotation. The ^1^H PCS differ by < 5% from the ^13^C PCS for PCS < 0.5 ppm and up to 10% for PCS larger than 0.5 ppm. Note that we neglected the effects of residual anisotropic chemical shifts and residual dipolar couplings due to partial alignment. In the plot, two exceptions can be seen. The angles of the metal–methyl vector of methyl groups L89 Cδ1 and V92 Cγ1 are very close to the angle where the PCS changes sign in mutant 149C/187C, resulting in a very high PCS gradient (Fig. 3). Thus, in general, the chemical shifts for the ^1^H and ^13^C are expected to be on a line with slope 0.95–1.05 for small PCS and 0.9–1.1 for large PCS, the correlation line. This property is used to identify which diamagnetic and paramagnetic resonances derive from the same nucleus and, thus, yield an experimental PCS (the pairing procedure). For well-dispersed parts of the spectrum pairing is trivial but in more crowded areas it can be ambiguous when more than one paramagnetic peak is on the correlation line. Pairing can either be done fully automatically with a script implemented in PARAssign based on these criteria, or in combination with manually paired peaks. When pairing manually, differences in peak intensity and shape can sometimes help (Fig. 4). Pairing is done iteratively, by pairing additional peaks using the predicted PCS after a run of PARAssign. In this way, 97% of ILV peaks were matched for both the mutants 50C/54C and 101C/105C and 58% of the mutant 149C/187C. The lower pairing level for the latter mutant as well as the relatively poor correlation between measured and calculated amide PCS suggest that the data for this mutant are of lower quality. It is unlikely that the tag causes a major structural modification because only small chemical shift differences between the spectra of the WT and Lu^3+^-CLaNP tagged mutant are observed (Fig. 2a). The tag is also connected via both arms, because the size of the Δχ-tensor is similar to that of the other mutants (Table S1). Single armed CLaNP yields strongly reduced Δχ-tensor values (Keizers et al. 2007). However, some unexpected broadening and the presence of double peaks in the paramagnetic spectrum suggest that the tag may assume more than one orientation relative to the protein. The tag in mutant 149C/187C CLaNP-5 crosslinks two β-strands, a type of attachment that has not been reported before, to the best of our knowledge. Perhaps this type of attachment results in some degree of conformational freedom of the tag. The Cα–Cα distance is about 1 Å shorter for this mutant than for the two other considered mutants. In the two other mutants, the CLaNP tag connects two consecutive turns on alpha-helices (Fig. 2b).

### A new criterion for reliability

**Fig. 5:**
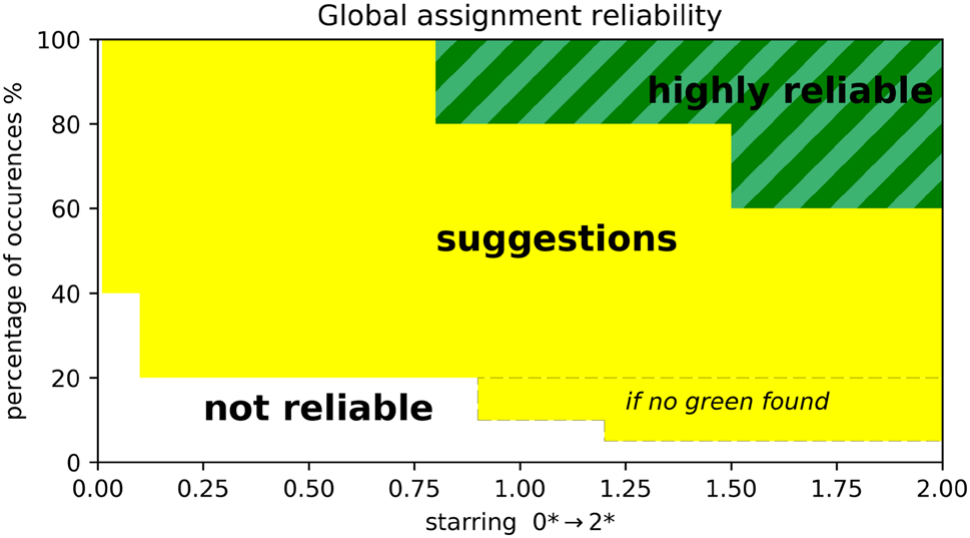
Assignment reliability. The average quality score from the 100 assignment runs is indicated on the horizontal axis and the occurrence percentage of the runs in which the assignment is found is plotted vertically. Dark and light green refer to stereospecific and non-stereospecific assignment of Leu and Val methyl groups

A consequence of the pairing problem is that the input data will, in general, not be complete, contrary to the synthetic data used in the simulation described in a previous paper (Skinner et al. 2013). To reduce possible artefacts in the assignment due to local minima in the search space, a jackknife procedure was implemented. Assignment is performed 100 times while leaving out 5% of the input PCS, randomly selected for each of the runs. During each run, PARAssign performs the assignment automatically using an iterative procedure. The back-calculated PCS are matched with the input PCS, followed by the optimization of the Δχ-tensor parameters and lanthanoid location. PARAssign assesses the reliability of the assignments with a starring system. If all (and at least two) PCS for a given methyl group are > 0.02 ppm and the combined fit of the PCS has a scoring factor (Eq. 2) lower than the average, the assignment is 2*. If only one of the criteria is met, the assignment gets 1*, otherwise 0*. After 100 runs, the fraction of occurrence (out of the 100 runs) of each assignment is combined with the average starring of the assignment to get an overall reliability, indicated with a color code, green, yellow and white for highly reliable, suggestion and not reliable, respectively, illustrated in Fig. 5. To be classified as highly reliable, colored in green, a residue assignment must occur in more than 60% of the runs with an average starring higher than 1.5 or occur more than 80% with an average starring higher than 0.8. Methyl groups that are assigned to only one of the two methyl groups in Val or Leu, i.e. methyls that are assigned stereo-specifically, are shown in dark green. If the residue is reliably assigned but both methyl groups are frequently identified as the correct assignment in the 100 consecutive assignment runs, the bar is shown in light green, indicating that the resonance has been assigned reliably but non-stereospecifically to a residue. It should be noted that the correctness of stereospecificity could not be assessed because no stereospecific assignments were available for ntd-HSP90. Assignments occurring in < 5% of the runs are deemed unreliable (white). For all the other assignments, colored in yellow, PARAssign finds one or more assignments with reasonable occurrence frequencies (higher than 40% but not already green, higher than 20% with a starring > 0.1, and, if no highly reliable assignment has been found, extra yellow assignments are proposed, see Fig. 5). Such a set of suggestions can still be very useful when combined with other strategies. Data from other sources (mutagenesis, NOEs) could be used to decide which of the assignments is the correct one.

### Methyl group assignment with simulated data

**Fig. 6:**
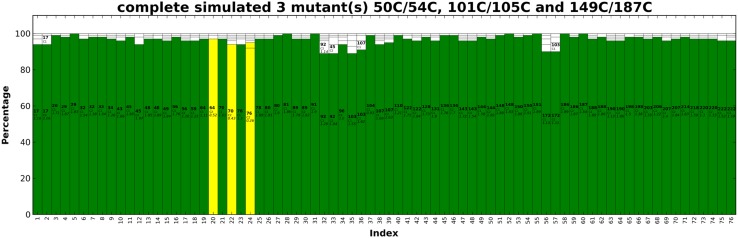
PARAssign output for a complete, simulated dataset. In green the highly reliable assignments and in yellow the suggested assignments are indicated. In each bar, the proposed residue and methyl group as well as the average starring value are shown. In this example, all the assignments are correct. See also the enlargement in Fig. 7

Ntd-HSP90 as represented in PDB entry 3T0Z contains 20 isoleucines labeled on the carbon δ1, 18 leucines labeled on both carbon δ1 and carbon δ2 and 10 valines labeled on both carbon γ1 and carbon γ2. Thus, in total 76 methyl groups need to be assigned. To establish whether PARAssign can solve the assignment problem, first the PCS for the 76 methyl groups were calculated using the Δχ-tensors for the three tag positions obtained from the amide PCS. PARAssign was run using the WT protein structure 3T0Z and the three simulated PCS datasets for input. The starting parameters of the three Δχ-tensors were Δχ_ax_ = 8.5 and Δχ_rh_ = 2.0. The starting angles and metal position were predicted by PARAssign purely based on reported positions relative to the Cys Cα and Cγ atoms and Δχ-tensor orientations for CLaNP-5, which has been shown to be a good approximation (Skinner et al. 2013; Keizers et al. 2008). Therefore, no prior experimental knowledge of the tensors is necessary. Using the simulated data for three paramagnetic centers as input, the vast majority of assignments was classified as highly reliable and all these ‘green’ assignments were correct. In addition, for the three assignments classified as ‘yellow’ the correct assignment was proposed (Table 1). The PARAssign output is displayed in Fig. 6. When using the data from only two tags, two of the three pairs of tags (50C/54C with 101C/105C and 101C/105C with 149C/187C) yield similar results as with three tags (Table 1, Fig. S2). Combining the data of mutants 50C/54C and 149C/187C yields mostly assignments with more than one possibility (‘yellow’), on the whole. The reason for this striking difference will be discussed below. We can conclude from the assignment runs with synthetic datasets that the assignment problem can be solved by PARAssign with complete and error-free data input from two or three tag positions.

**Table 1:**
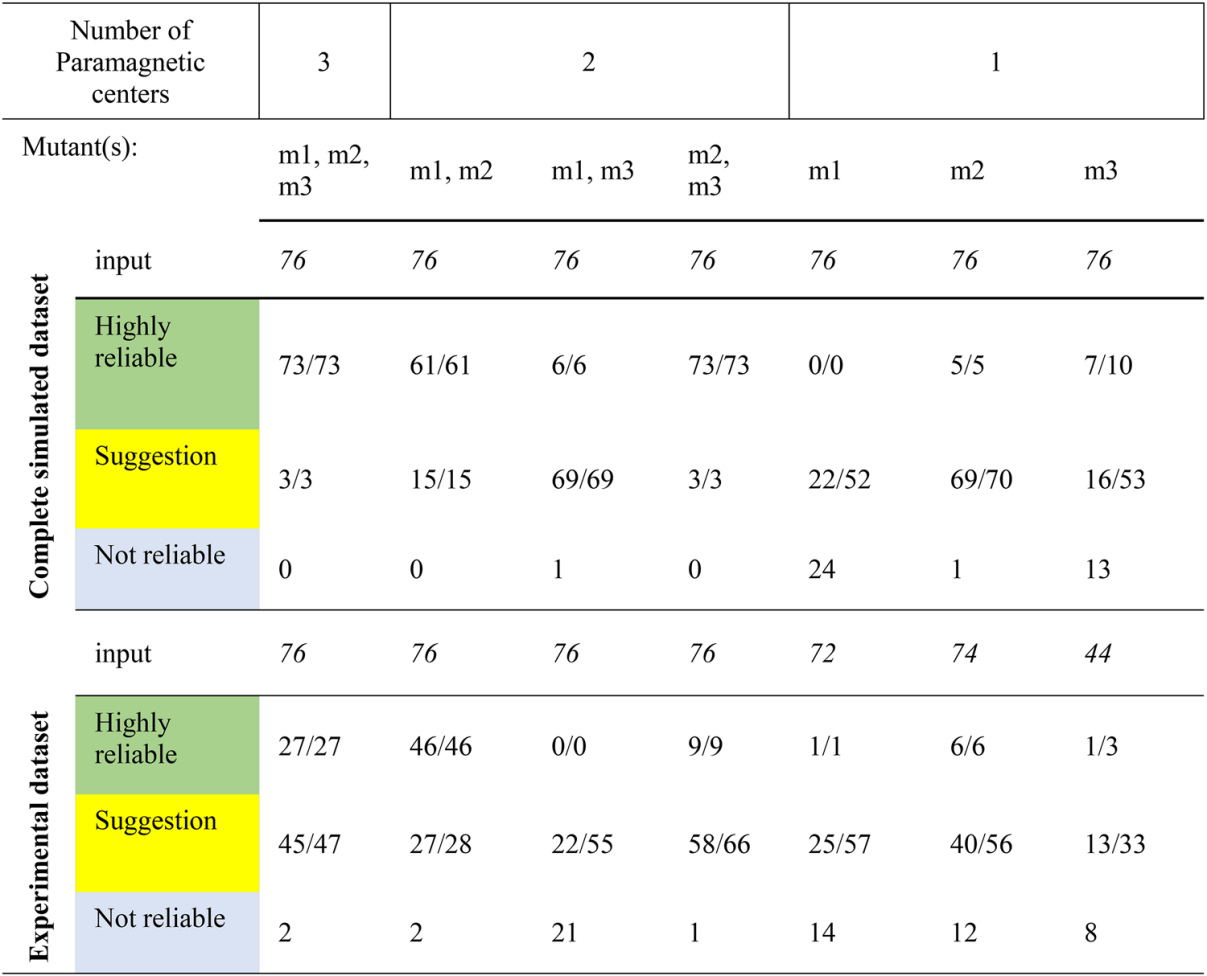
Assignment of the methyl groups

The terms m1, m2 and m3 refer to the mutants 50C/54C, 101C/105C and 149C/187C, respectively. For the green category, the numbers represent the correct/total number of assignments. For the yellow category, the first number indicates for how many residues the correct residue was among the assignment suggestions, the second represents the total number of assignments in this category

**Fig. 7:**
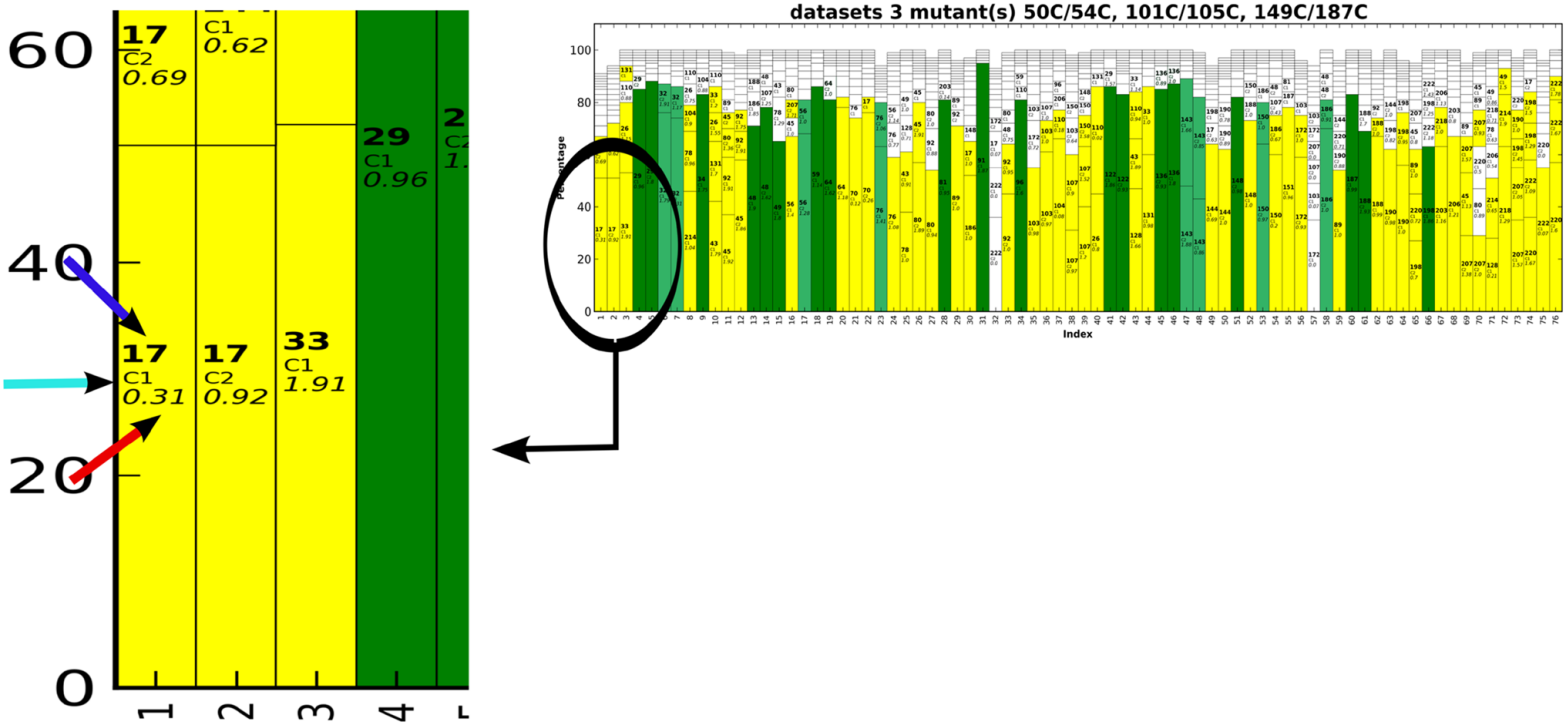
PARAssign output for the complete experimental dataset. The highly reliable assignments are shown in green and the proposed reliable assignments are in yellow. In this example, all the green assignments are correct and 95% of the yellow assignments contain the correct assignment (among 1–4 possibilities). In the close-up the blue, cyan and red arrows indicate the residue number, the stereospecificity and the scoring factor, respectively

### Assignment of methyl groups with experimental datasets

**Fig. 8:**
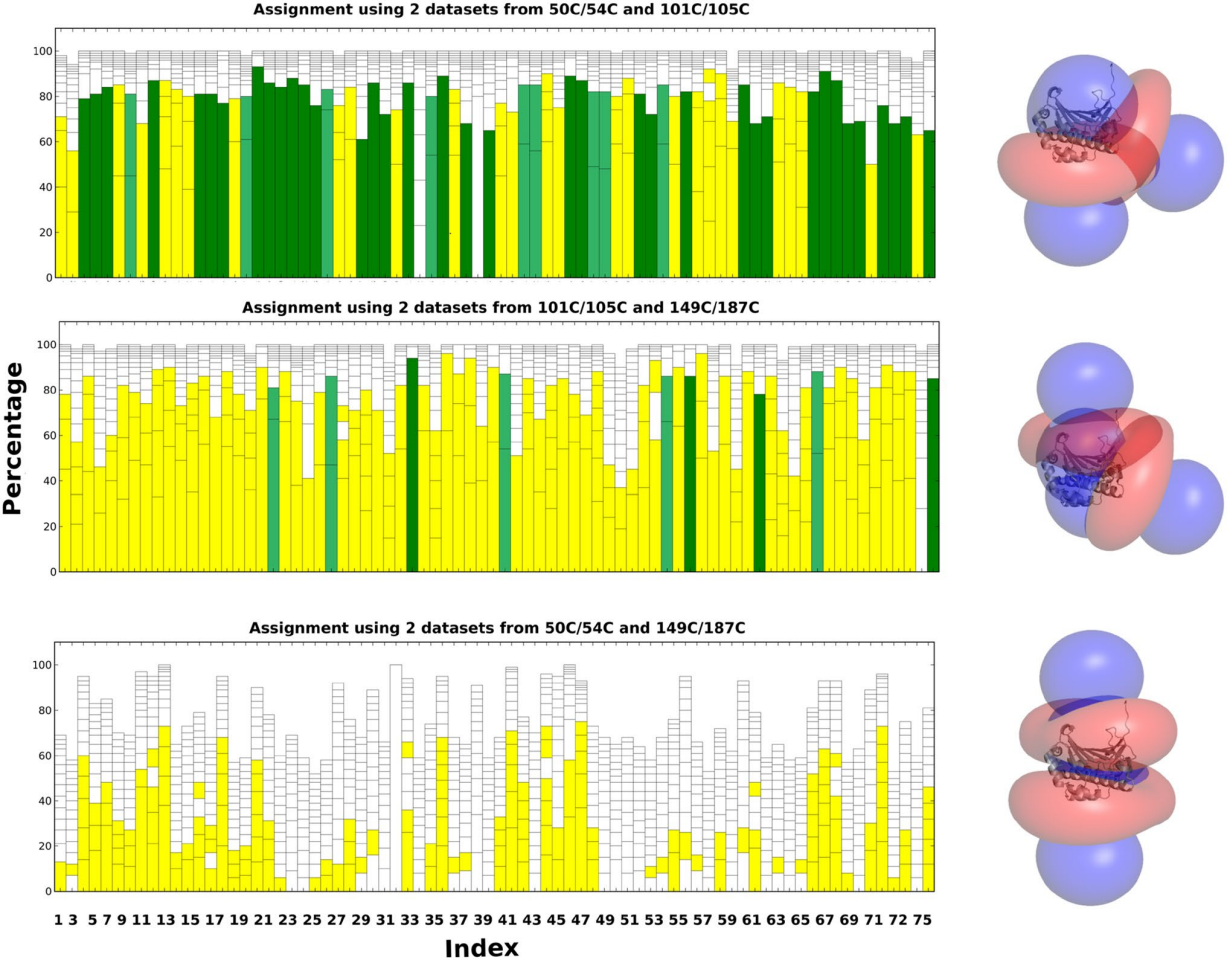
PARAssign output for the three combinations of two of the experimental data sets. Labels were removed for clarity. On the right the corresponding relative orientations of the two CLaNP-5 tensors are shown

First, the methyl resonances of ntd-HSP90 were assigned using experimental datasets from the three paramagnetic centers. The PCS pairing procedure yielded 72, 74 and 44 of the expected 76 PCS for the mutants 50C/54C, 101C/105 and 149C/187C, respectively. As we had collected the spectra of Ile labeled protein samples separately from those labeled with Leu and Val (see “Materials and methods”), it was possible to identify Ile methyl groups but we could not distinguish Leu and Val methyl groups. Therefore, in the input file the two possible residue types were entered as “LEU VAL”. In case of other methyl group labeling schemes, the residue type can be entered with its amino acid code, for example, “MET” for the methionine methyl group labeling. PARAssign runs were performed as described above, by using either the data from three, two, or one paramagnetic center(s). The results are listed in Table 1 and PARAssign output using experimental datasets of three mutants is displayed Fig. 7. The tensor parameters are given in Table S2 and Figs. S3, S4. In the results based on three datasets, all of the 27 highly reliable assignments were correct and 95% of the yellow assignments contained the correct assignment (1–4 possibilities). The assignment procedure was also performed with the input data of only two of the three paramagnetic centers (Fig. 8). In each case, all of the highly reliable (green) assignments were correct. Interestingly, however, the number of highly reliable assignments was very different for the three different combinations of paramagnetic centers. With the combination (50C/54C–101C/105C), 46 methyl groups were assigned with high reliability, whereas only nine assignments were highly reliable for the combination (101C/105C–149C/187C) and none for the combination (50C/54C–149C/187C). Furthermore, this combination yielded very few reliable (yellow) assignments. The much smaller input data set for mutant 149C/187C partly explains why fewer reliable assignments were obtained for combinations with this mutant. However, the large difference in assignment quality between the two combinations that contain the 149C/187C data must have another reason. We ascribe this to the relative orientations of the Δχ-tensors (Fig. 8). For parallel Δχ-tensors the assignment quality is poorer than for orthogonal Δχ-tensors, which was confirmed by a run of PARAssign with complete, simulated datasets for which the combination 50C/54C–149C/187C yielded many fewer highly reliable assignments than the two other combinations (Table 1).

**Fig. 9:**
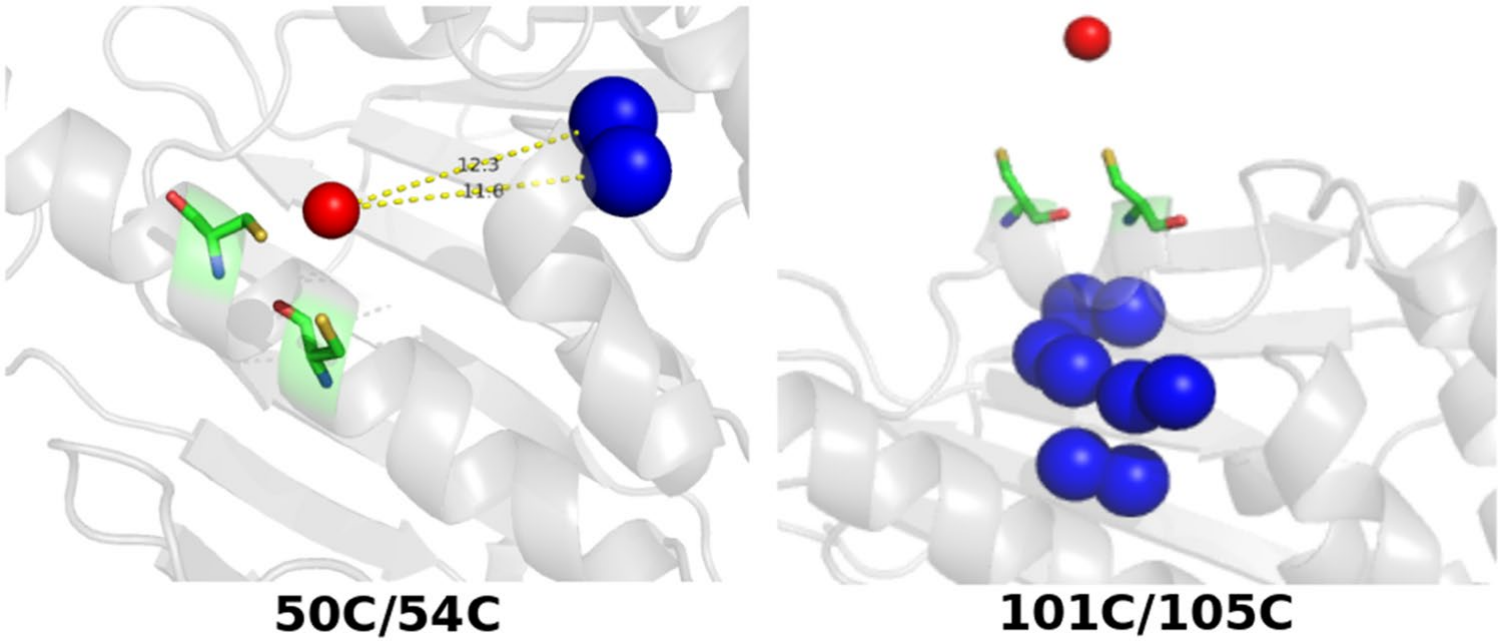
Highly reliably assigned methyl groups, represented in blue spheres, using a single paramagnetic center for the assignment. The numbers in the 50C/54C panel indicate the distances from the lanthanoid to the methyl groups in Å

The assignment runs were also performed using data from only one paramagnetic center. As expected, the number of correct assignments drops dramatically. Nevertheless, PARAssign identified 1–6 highly reliable assignments for the two mutants 50C/54C and 101C/105C, which were all correct. These assignment calculations were performed using a PRE cutoff of 9 Å (Eq. 1) to prevent methyl groups for which the PRE leads to extensive broadening, being considered for assignment. The 149C/187C mutant did not yield correct assignments because of the limited input set due to low quality of the spectra. Therefore, when using PARAssign with only one dataset, the quality of the input becomes essential to avoid false positive assignments. From this study, we conclude that with a dataset containing < 60% of the expected PCS, PARAssign fails to give highly reliable assignments that are all correct. The highly reliably assigned nuclei from the two mutants with good input data correspond to methyl groups that are localized close to the lanthanoid, displaying sizeable PCS, as illustrated in Fig. 9. Thus, the use of a single tag could be considered for localized assignment, for instance of methyl groups in an active site.

The quality of the assignment is reflected in the accuracy and precision of the Δχ tensors parameters refined by PARAssign. The sinusoidal projection and histogram representation of the Euler angles of refined tensors are shown in Figs. S3 and S4, respectively. The combination (mutant 50C/54C–101C/105C) leading to the best assignments is also the one for which tensor orientations are the best refined. This is to be expected, because high reliability is obtained only if the same assignments are found consistently over the 100 runs. The combinations of data sets that lead to poor assignment reliability also yield a large spread in the Euler angles, indicating a poor reproducibility of tensor determination over the 100 runs. A comparison of the precision of the 50C/54C tensor calculated when using the single data set as compared to the combination with 149C/C187C shows that the former is higher. This analysis strongly suggests that the addition of a relatively poor dataset of a parallel Δχ-tensor can worsen the assignment process.

## Conclusions

The results presented in this study demonstrate possibilities and limitations for use of PCS in the assignment of protein methyl group resonances. The simulations show that, with complete input, the search problem can be solved with data from three and even from only two paramagnetic tags. The quality of the input data is vitally important. One of the three tags yielded broader peaks and poorer spectra, resulting in a significant decrease in the number of paramagnetic peaks that could be paired with their diamagnetic counterparts. Such an incomplete PCS data set strongly affects the quality of the assignment. In our hands, CLaNP attached to two consecutive turns of an α-helix gives the best results with minimal disturbance of the protein structure. It was also found that the Δχ tensors of different tags should not be oriented along the same main axis, because this results in correlated PCS and reduced assignment quality. These factors can easily be taken into account when designing the tag positions. With two-armed tags such as CLaNP-5, the lanthanoid location and approximate directions of the axes of the Δχ tensor can be estimated well. It is then possible to predict the PCS for different tags and use these in PARAssign to predict whether complete assignment will be achieved if good spectra can be acquired.

PARAssign version 2 runs multiple times with a small part of the input data removed to yield more robust assignments. It was demonstrated that, with data from two or three independent tags, assignments classified as highly reliable are correct and that in those classified as ‘yellow’, the correct assignment is nearly always among the few suggested possibilities. Such information could be combined with data from NOE, PRE or mutagenesis experiments to find a unique assignment.

The critical point in obtaining complete PCS input sets is the pairing of peaks in the paramagnetic and diamagnetic spectra. Clearly, the larger the number of peaks in a spectrum, the more complicated this process is, in particular for methyl resonances, which show little dispersion. For larger systems, preparing several samples with only one type of amino acid labeled will reduce the overlap (Pederson et al. 2017; Zhang and Ingen 2016; Miyanoiri et al. 2016). Labeling a single methyl group per amino acid also helps (Tugarinov et al. 2006). All the PCS can then be combined in PARAssign, with the amino acid types indicated. Another approach to assist the pairing of peaks is to prepare a sample with the same tag containing a weakly paramagnetic lanthanoid, such as Eu^3+^. The Δχ tensors of different lanthanoids in the same tag are oriented to good approximation in the same direction, so that a weaker lanthanoid results in a resonance on the correlation line between the diamagnetic and paramagnetic peak, at a position determined by the ratio of the sizes of the Δχ tensors of the stronger and weaker lanthanoids. With Eu^3+^ the PCS are small and more readily attributable to the corresponding diamagnetic peak.

If some assignments are already known, these can be introduced in PARAssign to improve the assignment results. To confirm the correctness of the available assignments of the methyl resonances in ntd-HSP90, we mutated L45, L80 and L89 one by one to Ile. When these assignments were fixed in the PARAssign calculations, the number of highly reliable assignments improved by up to 15% for the best combination.

A single tag cannot be used to obtain an overall assignment, because of the symmetric nature of the paramagnetic effect, in particular for axial tensors. However, in combination with the protein structure, resonances of nearby methyl groups can still be assigned. This approach can be helpful to highlight a certain region of a protein, for example the active site in an enzyme. The assigned nuclei can then be used to report on dynamics or structural changes that occur in its proximity.

## Electronic supplementary material

Below is the link to the electronic supplementary material.

Supplementary material 1 (DOCX 3861 KB)

## Abbreviations

PCS: Pseudocontact shift
RDC: Residual dipolar coupling
PRE: Paramagnetic relaxation enhancement
HSQC: Heteronuclear single quantum coherence
TROSY: Transverse relaxation optimized spectroscopy
CLaNP-5: Caged lanthanoid NMR probe 5
ntd-HSP90: N-terminal domain of heat shock protein 90

## References

[CR1] Amero C (2011). A systematic mutagenesis-driven strategy for site-resolved NMR studies of supramolecular assemblies. J Biomol NMR.

[CR2] Bertini I, Luchinat C, Parigi G, Ravera E (2015). NMR of paramagnetic molecules.

[CR3] Chao FA (2014). FLAMEnGO 2.0: an enhanced fuzzy logic algorithm for structure-based assignment of methyl group resonances. J Magn Reson.

[CR4] Delaglio F (1995). NMRpipe—a multidimensional spectral processing system based on UNIX pipes. J Biomol NMR.

[CR5] Gelis I (2007). Structural basis for signal-sequence recognition by the translocase motor SecA as determined by NMR. Cell.

[CR6] Gross JD, Gelev VM, Wagner G (2003). A sensitive and robust method for obtaining intermolecular NOEs between side chains in large protein complexes. J Biomol NMR.

[CR7] Jacobs DM (2006). NMR backbone assignment of the N-terminal domain of human HSP90. J Biomol NMR.

[CR8] John M (2007). Sequence-specific and stereospecific assignment of methyl groups using paramagnetic lanthanides. J Am Chem Soc.

[CR9] Karagoz GE (2011). N-terminal domain of human Hsp90 triggers binding to the cochaperone p23. Proc Natl Acad Sci USA.

[CR10] Keizers PH, Desreux JF, Overhand M, Ubbink M (2007). Increased paramagnetic effect of a lanthanide protein probe by two-point attachment. J Am Chem Soc.

[CR11] Keizers PHJ, Saragliadis A, Hiruma Y, Overhand M, Ubbink M, Design (2008). Synthesis, and evaluation of a lanthanide chelating protein probe: CLaNP-5 yields predictable paramagnetic effects independent of environment. J Am Chem Soc.

[CR12] Kraft D (1988) A software package for sequential quadratic programming (trans: Center DGA). Tech Rep. DFVLR-FB 88–28. Institute for Flight Mechanics, Koln

[CR13] Kuhn HW (1955). The Hungarian method for the assignment problem. Nav Res Logist.

[CR14] Li J, Soroka J, Buchner J (2012). The Hsp90 chaperone machinery: conformational dynamics and regulation by co-chaperones. BBA-Mol Cell Res.

[CR15] Li J (2012). Structure insights into mechanisms of ATP hydrolysis and the activation of human heat-shock protein 90. Acta Biochem Biophys Sin.

[CR16] Mas G, Crublet E, Hamelin O, Gans P, Boisbouvier J (2013). Specific labeling and assignment strategies of valine methyl groups for NMR studies of high molecular weight proteins. J Biomol NMR.

[CR17] Miyanoiri Y (2016). Highly efficient residue-selective labeling with isotope-labeled Ile, Leu, and Val using a new auxotrophic E-coli strain. J Biomol NMR.

[CR18] Pan YZ (2016). Sequence-specific assignment of methyl groups from the neuronal SNARE complex using lanthanide-induced pseudocontact shifts. J Biomol NMR.

[CR19] Pearl LH (2016). The HSP90 molecular chaperone—an enigmatic ATPase. Biopolymers.

[CR20] Pederson K (2017). NMR characterization of HtpG, the *E. coli* Hsp90, using sparse labeling with 13C-methyl alanine. J Biomol NMR.

[CR21] Pervushin K, Riek R, Wider G, Wuthrich K (1997). Attenuated T2 relaxation by mutual cancellation of dipole-dipole coupling and chemical shift anisotropy indicates an avenue to NMR structures of very large biological macromolecules in solution. Proc Natl Acad Sci USA.

[CR22] Pintacuda G (2004). Fast structure-based assignment of N-15 HSQC spectra of selectively N-15-labeled paramagnetic proteins. J Am Chem Soc.

[CR23] Scheich C, Niesen FH, Seckler R, Bussow K (2004). An automated in vitro protein folding screen applied to a human dynactin subunit. Protein Sci.

[CR24] Schmitz C (2006). Efficient chi-tensor determination and NH assignment of paramagnetic proteins. J Biomol NMR.

[CR25] Schmitz C, Stanton-Cook MJ, Su XC, Otting G, Huber T (2008). Numbat: an interactive software tool for fitting Delta chi-tensors to molecular coordinates using pseudocontact shifts. J Biomol NMR.

[CR26] Shah DM (2012). Rapid protein-ligand costructures from sparse NOE data. J Med Chem.

[CR27] Skinner SP, Moshev M, Hass MA, Keizers PH, Ubbink M (2013). PARAssign—paramagnetic NMR assignments of protein nuclei on the basis of pseudocontact shifts. J Biomol NMR.

[CR28] Sprangers R, Kay LE (2007). Quantitative dynamics and binding studies of the 20S proteasome by NMR. Nature.

[CR29] Tugarinov V, Kay LE (2005). Methyl groups as probes of structure and dynamics in NMR studies of high-molecular-weight proteins. ChemBioChem.

[CR30] Tugarinov V, Hwang PM, Ollerenshaw JE, Kay LE (2003). Cross-correlated relaxation enhanced 1H[bond]13C NMR spectroscopy of methyl groups in very high molecular weight proteins and protein complexes. J Am Chem Soc.

[CR31] Tugarinov V, Kanelis V, Kay LE (2006). Isotope labeling strategies for the study of high-molecular-weight proteins by solution NMR spectroscopy. Nat Protoc.

[CR32] Velyvis A, Schachman HK, Kay LE (2009). Assignment of Ile, Leu, and Val methyl correlations in supra-molecular systems: an application to aspartate transcarbamoylase. J Am Chem Soc.

[CR33] Venditti V, Fawzi NL, Clore GM (2011). Automated sequence- and stereo-specific assignment of methyl-labeled proteins by paramagnetic relaxation and methyl-methyl nuclear overhauser enhancement spectroscopy. J Biomol NMR.

[CR34] Vranken WF (2005). The CCPN data model for NMR spectroscopy: development of a software pipeline. Proteins.

[CR35] Wiesner S, Sprangers R (2015). Methyl groups as NMR probes for biomolecular interactions. Curr Opin Struct Biol.

[CR36] Xu YQ, Matthews S (2013). MAP-XSII: an improved program for the automatic assignment of methyl resonances in large proteins. J Biomol NMR.

[CR37] Zhang HY, van Ingen H (2016). Isotope-labeling strategies for solution NMR studies of macromolecular assemblies. Curr Opin Struct Biol.

