# Supplementary material for: Methyl group assignment using pseudocontact shifts with PARAssign
Source: J Biomol NMR. 2017 Nov 27;69(4):183–95. doi: 10.1007/s10858-017-0136-3 (PMC5736784; doi:10.1007/s10858-017-0136-3)
Supplement: Supplementary file 1 — Supplementary material 1 (DOCX 3861 KB) [file 10858_2017_136_MOESM1_ESM.docx]

Supplementary material to

**Methyl group assignment using pseudocontact shifts with PARAssign**

Mathilde Lescanne, Simon P. Skinner, Anneloes Blok, Monika Timmer, Linda Cerfolini, Marco Fragai, Claudio Luchinat, and Marcellus Ubbink.

**Table S1** Susceptibility Δχ-tensors obtained by fitting amide PCS of ntd-HSP90 (PDB entry 3T0Z ^[29](#_ENREF_29)^).

| **Mutant tagged with Yb^3+^-CLaNP-5** | **50C/54C** | **101C/105C** | **149C/187C** |
| --- | --- | --- | --- |
| Δχ_ax_ (10^-32^ m^3^) | 8.4 (0.1) | 8.0 (0.2) | 8.3(0.3) |
| Δχ_rh_ (10^-32^ m^3^) | 2.0 (0.3) | 1.6(0.5) | 2.2(0.3) |
| α (°) | 173(5) | 180(2) | 176(3) |
| β (°) | 155(1) | 88(2) | 148(1) |
| γ (°) | 150(3) | 170(3) | 15(4) |
| x (Å) | 12.4(0.5) | 25.3(0.2) | -1.8(0.2) |
| y (Å) | 1.3(0.5) | -9.5(0.2) | -5.8(0.5) |
| z (Å) | 25.2(0.3) | 6.8(0.2) | -1.2(0.2) |
| Restraints | 80 | 63 | 69 |
| Q value* | 0.056 | 0.068 | 0.26 |
| Q^a^ value* | 0.028 | 0.034 | 0.12 |
| Average pcs deviation (ppm) | 0.012 | 0.009 | 0.035 |

* The Q-values were calculated using:

$$Q=\sqrt{\frac{\sum\left( \delta_{PCS,i}^{pred}-\delta_{PCS,i}^{exp} \right)^{2}}{\sum\left( \delta_{PCS,i}^{exp} \right)^{2}}}$$

$$Q^{a}=\sqrt{\frac{\sum\left( \delta_{PCS,i}^{pred}-\delta_{PCS,i}^{exp} \right)^{2}}{\sum\left( \left| \delta_{PCS,i}^{pred} \right|+\left| \delta_{PCS,i}^{exp} \right| \right)^{2}}}$$

**Table S2** Tensor parameters refined for the different combinations of datasets. In parenthesis are the standard deviations. The Euler angles α, β, γ are defined according to the z-y-z convention. A * indicates that there are other conformations. See also Figures S3 and S4.

|  | 3 paramagnetic centers | | | 2 paramagnetic centers | | | | | | 1 paramagnetic center | | |
| --- | --- | --- | --- | --- | --- | --- | --- | --- | --- | --- | --- | --- |
|  |  |  |  | 50C/54C – 101C/105C | | 50C/54C – 149C/187C | | 101C/105C – 149C/187C | |  |  |  |
|  | 50C/54C | 101C/105C | 149C/187C | 50C/54C | 101C/105C | 50C/54C | 149C/187C | 101C/105C | 149C/187C | 50C/54C | 101C/105C | 149C/187C |
| Δχ_ax_  (10^-32^ m^3^) | 8.1 (0.7) | 7.5 (0.6) | 8.2 (0.9) | 7.8 (0.5) | 7.7 (0.7) | 7.6 (0.8) | 7.2 (0.5) | 7.6 (0.6) | 8.5 (0.8) | 7.3 (0.7) | 7.3 (0.5) | 8.3 (0.6) |
| Δχ_rh_  (10^-32^ m^3^) | 2.0 (0.4) | 2.4 (0.3) | 2.4 (0.3) | 2.1 (0.4) | 2.4 (0.2) | 2.2 (0.4) | 2.4 (0.3) | 2.0 (0.3) | 2.2 (0.3) | 2.4 (0.2) | 2.0 (0.4) | 2.2 (0.3) |
| α (°) | -144 (9)* | -177 (12) | 15 (81) | -38 (5) | -169 (15) | -113 (28) | -2 (38) | -174 (16)* | -47 (15) | 118 (24) | -168 (6)* | 44 (12) |
| β (°) | 146 (9) | 103 (8) | 142 (11) | 31 (5) | 96 (8) | 64 (18) | 97 (38) | 104 (6) | 151 (7) | 30 (4) | 98 (4) | 153 (6) |
| γ (°) | 21 (6) | 176 (6) | 19 (16) | -160 (5) | 176 (8) | -136 (23) | 159 (10)* | 176 (10) | 6 (7) | -142 (14) | 160 (2)* | -1 (14) |
| x (Å) | 14.0 (2.3) | 22.1 (5.4) | -3.8 (3.6) | 13.0 (1.3) | 23.8 (3.1) | 14.9 (2.7) | -2.3 (6.2) | 24.9 (0.4) | -1.6 (2.2) | 11.2 (1.7) | 25 (0.4) | -4.9 (1.3) |
| y (Å) | -1.6 (2.6) | -9.0 (4.3) | -4.4 (2.7) | -1.0 (1.4) | -8.8 (3.1) | -2.4 (2.6) | -4.5 (6.5) | -7.4 (1.3) | -5.8 (1.4) | -1.6 (1.1) | -6.6 (1.7) | -8.4 (1.7) |
| z (Å) | 24.4 (1.9) | 4.2 (2.9) | 0.1 (1.6) | 24.5 (1.9) | 6.3 (2.7) | 22.3 (3.3) | -0.2 (5.1) | 4.8 (1.3) | -1.4 (1.0) | 25.6 (0.7) | 5.6 (1.1) | -0.2 (1.0) |

**Fig. S1** Quality of Δχ fits. The calculated PCS are plotted against the observed PCS of amide groups of ntd-HSP90 tagged with Yb^3+^-CLaNP-5 at the indicated positions. The solid lines represent a perfect correlation (x=y).

**
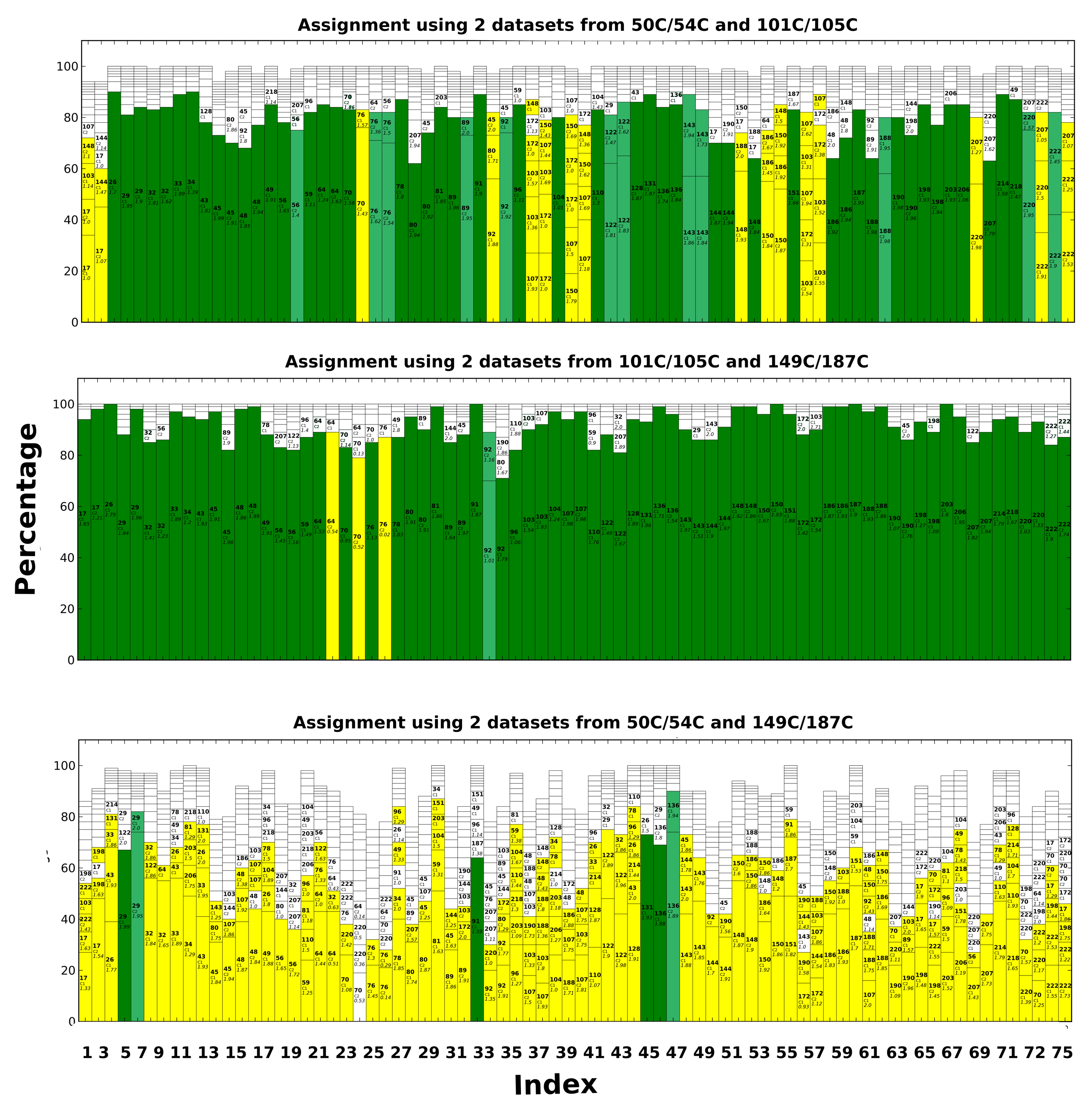
**

**Fig. S2** PARAssign output for the three combinations of two of the complete, simulated datasets.

**
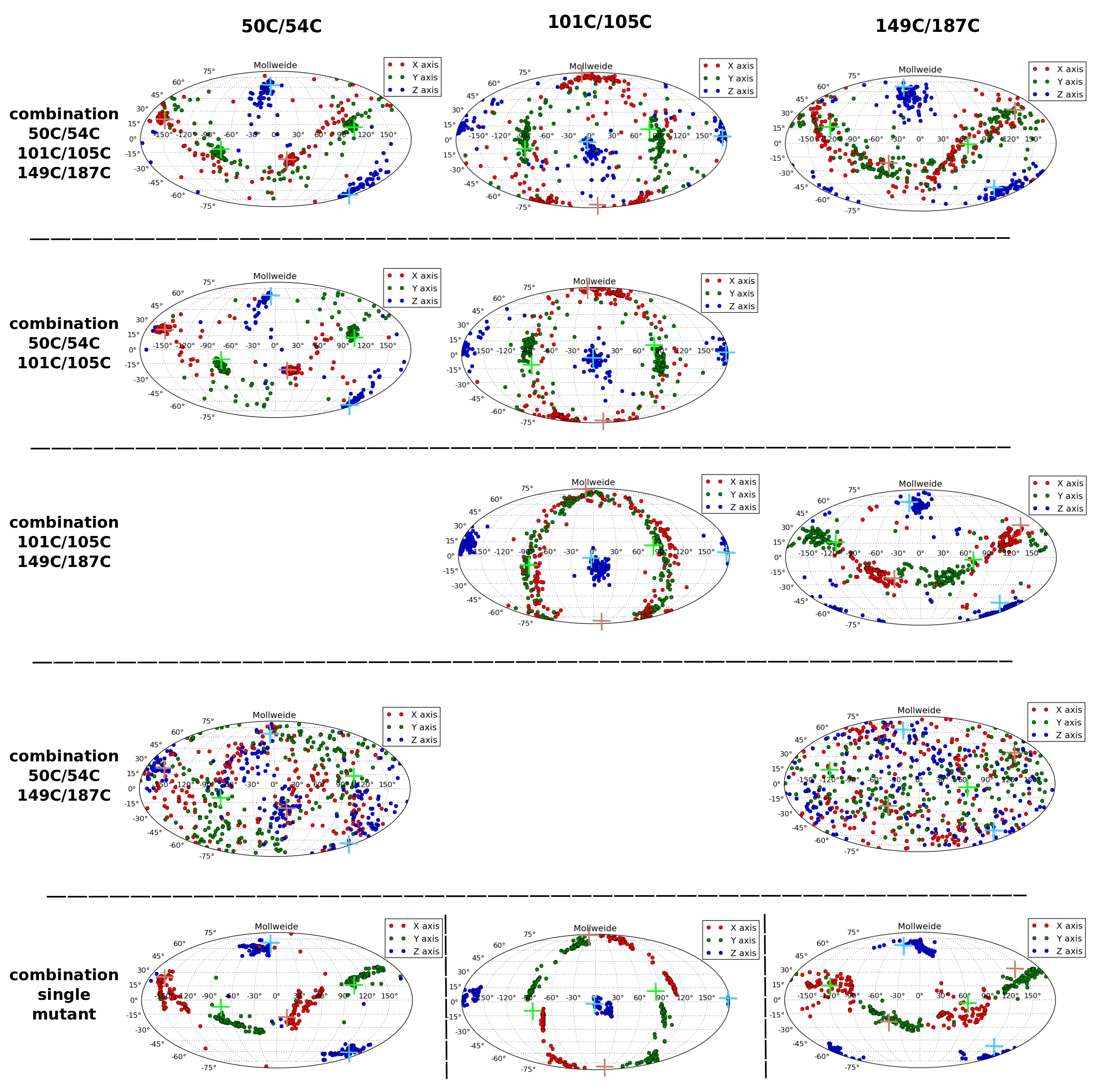
**

**Fig. S3** Mollweide sinusoidal representation of the tensor axes refined with PARAssign for all 7 combinations. The light crosses are the axis coordinates of the tensors refined with amide PCS, Table S1. A clear correlation can be observed between the number of highly reliable assignments for a given correlation and the quality of the tensor refinement.


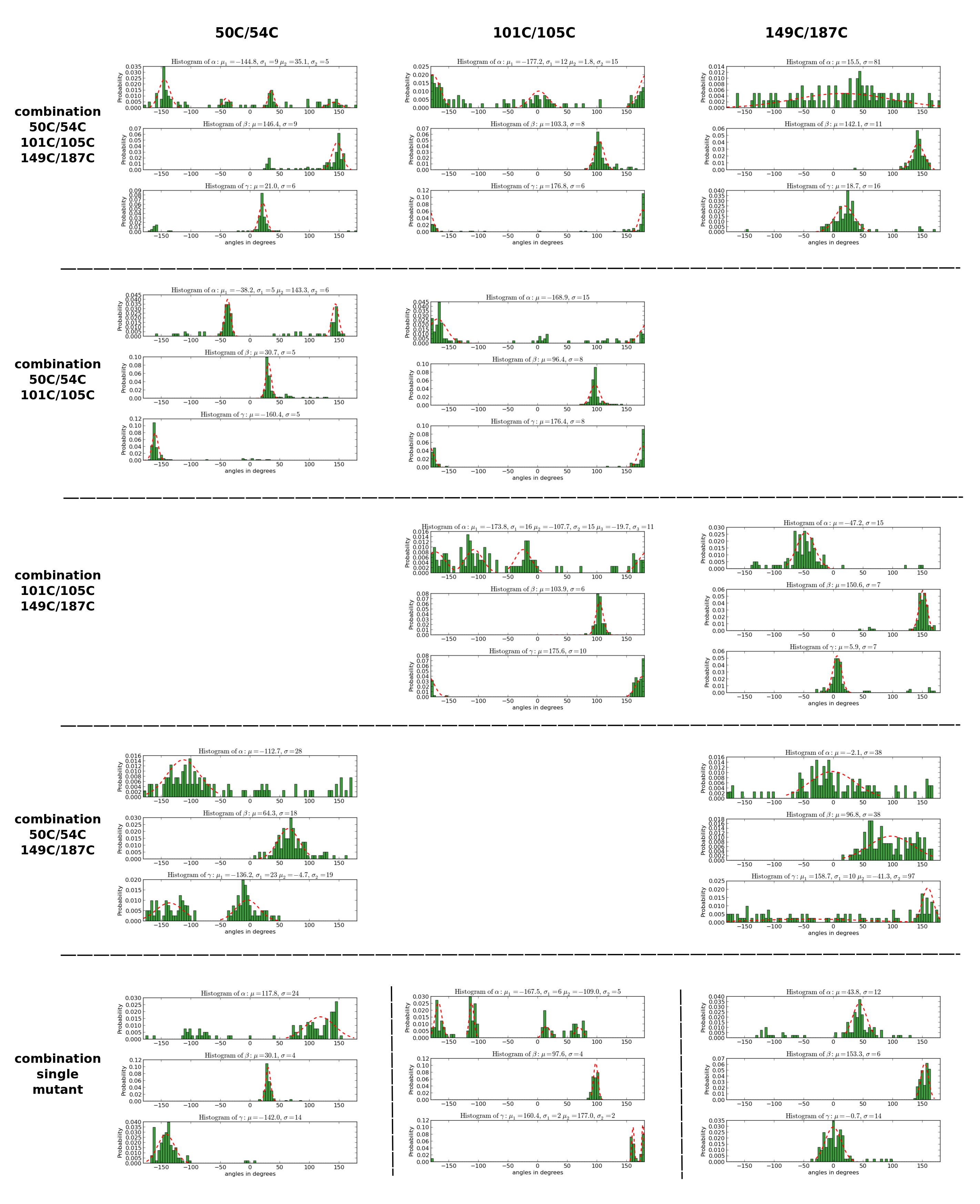


**Fig S4.** Distribution of Δχ Euler angles (convention z-y-z) for the 7 combinations. For all angle sets, the top, middle and bottom subplots are the histograms of α, β and γ, respectively. It should be noted that the zyz Euler representation (α, β, γ) $\setminus\in[0, 180[$is equivalent to (α+180, -β, -γ)$\setminus\in[-180, 0[$ zyz Euler representation. Additionally, the xz and yz planar symmetries of Δχ lead to equivalence by 180° rotation.
